# Characterizing the Social Epigenome in Mexican Patients with Early-Onset Psychosis

**DOI:** 10.3390/genes16050591

**Published:** 2025-05-17

**Authors:** David Ruiz-Ramos, José Jaime Martínez-Magaña, Isela Esther Juárez-Rojop, Germán Alberto Nolasco-Rosales, Fernanda Sosa-Hernández, Juan Daniel Cruz-Castillo, Josefa Cavazos, Adriana Callejas, Patricia Zavaleta-Ramírez, José Antonio Zorrilla-Dosal, Nuria Lanzagorta, Humberto Nicolini, Janitza L. Montalvo-Ortiz, David C. Glahn, Alma Delia Genis-Mendoza

**Affiliations:** 1 Academic Division of Health Sciences, Juárez Autonomous University of Tabasco (UJAT), Villahermosa 86100, Mexico; daruiz_914@hotmail.com (D.R.-R.); iselajuarezrojop@hotmail.com (I.E.J.-R.); ganr_1277@live.com.mx (G.A.N.-R.); juandaniel881@gmail.com (J.D.C.-C.); 2 Department of Psychiatry, Yale School of Medicine, Yale University, New Haven, CT 06520, USA; jose.martinez-magana@yale.edu (J.J.M.-M.); janitza.montalvo-ortiz@yale.edu (J.L.M.-O.); 3 VA Connecticut Healthcare System, West Haven, CT 06516, USA; 4 U.S. Department of Veterans Affairs National Center for Posttraumatic Stress Disorder, Clinical Neurosciences Division, West Haven, CT 06516, USA; 5 Dr. Juan N. Navarro Children’s Psychiatric Hospital, National Commission on Mental Health and Addictions (CONASAMA), Ministry of Health, Mexico City 14080, Mexico; fer.sosaher98@gmail.com (F.S.-H.); josefa.cavazos@gmail.com (J.C.); callejamta@hotmail.com (A.C.); zavaletarp@gmail.com (P.Z.-R.); psiquiatrajosezorrilla@gmail.com (J.A.Z.-D.); 6 Carracci Medical Group, Department of Clinical Research, Mexico City 03740, Mexico; lanzagorta_nuria@gmc.org.mx (N.L.); hnicolini@inmegen.gob.mx (H.N.); 7 Genomics Laboratory of Psychiatric, Neurodegenerative, and Addiction Disorders, National Institute of Genomic Medicine (INMEGEN), Ministry of Health, Mexico City 14610, Mexico; 8 Department of Psychiatry and Behavioral Sciences, Boston Children’s Hospital, Boston, MA 02115, USA; david.glahn@childrens.harvard.edu; 9 Department of Psychiatry, Harvard Medical School, Boston, MA 02215, USA; 10 Olin Neuropsychiatry Research Center, Institute of Living, Hartford, CT 06106, USA

**Keywords:** early-onset psychosis, epigenome-wide association study, methylation risk score, Mexico, adolescents

## Abstract

**Background:** Psychosis is one of the leading causes of disability worldwide. Individuals with early-onset psychosis (EOP) tend to experience a worse prognosis and shorter life expectancy. The etiology of EOP remains unclear, but epigenetic mechanisms are known to serve as the interface between environmental exposures and biological processes to better understand its etiology. **Objectives:** We characterized the sociodemographic and clinical characteristics, as well as genome-wide epigenetic markers, in Mexican patients with EOP. **Methods:** We estimated epigenetic age, performed an epigenome-wide association study, and finally developed an epigenetic risk score (MRS) to predict manifestations of psychosis. **Results:** We found that patients with EOP have a higher epigenetic age using Wu’s clock (*p *= 0.015). Moreover, accelerated epigenetic age was correlated with chronological age (PedBE clock, *p *= 0.046), global functioning (Wu’s clock, *p *= 0.027), and psychiatric admissions (DNAmTL, *p *= 0.038). In addition, we observed that a reduction in years of schooling is associated with an increase on epigenetic age (Levine’s clock, β = 5.07, *p *= 0.001). In our epigenome-wide association study, we identified eight CpGs associated with EOP. Noteworthy, a psychosis-methylation risk score (EOP-MRS) was associated with panic disorder (β = 1.36, *p *= 0.03), as well as auditory (β = 1.28, *p *= 0.04) and visual (β = 1.22, *p *= 0.04) hallucinations. **Conclusions:** Years of education have an impact on epigenetic age. Additionally, our study suggests associations of DNA methylation with EOP. Finally, we developed an MRS that associates clinical manifestations of psychosis.

## 1. Introduction

Early-onset psychosis (EOP) is a mental disorder characterized by the onset of psychosis before the age of 18 years [1]. Compared with adult-onset psychosis, those affected by EOP have shorter life expectancy, and poor treatment response [2,3]. The prevalence of psychotic symptoms has been reported to be higher in adolescents than in adults, with an estimated of 8–17% in children and adolescents [4,5,6], but there still need more evidence to support this.

The etiology of EOP is still unknown. Authors have proposed that abnormal neurodevelopment and early neurodegeneration explain the emergence of psychosis in the young population [7,8]. Furthermore, the literature suggests that environmental factors such as bullying, cannabis use, tobacco use, low birth weight, and childhood trauma interact with biological factors in the development of psychosis [3,9,10,11,12]. On the other hand, DNA methylation (DNAm), proposed to be a mediator between environmental exposures and biological effects, is the most studied epigenetic mechanism and could provide a better understanding of biological mechanisms underlying psychotic disorders [13]. New evidence from epigenome-wide association studies (EWASs), a comparison of DNAm sites across the genome, revealed associations with schizophrenia and first episode of psychosis (FEP) [14,15]. Additionally, DNAm is associated with psychotic symptoms in adults and the risk of neuropsychiatric disorders during childhood [8,16]. However, an EWAS of clinically defined EOP has not been performed.

The development of novel biomarkers derived from DNAm provides a new approach to understanding disease risk and biological and etiological mechanisms, such as aging [17,18,19]. For example, epigenetic clocks are excellent biomarkers to estimate biological age, also referred to as epigenetic age [7]. To date, three studies have shown evidence that epigenetic age correlates with the severity of psychosis, and accelerated biological age is associated with psychotic disorders [20,21,22,23]. Furthermore, the development of methylation risk scores (MRSs), representing the sum of an individual epigenetic risks derived from EWAS results, a similar construct of polygenic risk score, have been associated with schizophrenia [24], FEP [17], and neuroimaging changes in individuals with psychosis [8,25]. Studies using MRS showed that it could be used to differentiate individuals affected by psychosis and mediation effects of childhood adversity to develop psychosis risk [15]. It is noteworthy that, like in many biomedical research areas, there is still a lack of diversity in EWASs [26].

The current understanding of EOP is still limited and there is scare evidence of epigenetic biomarkers, to advance in these field, the current study aimed to characterize for the first time sociodemographic and clinical characteristics, with a further comprehensive evaluation of genome-wide epigenetic markers (including 11 epigenetic clocks and EOP-MRS) in Mexican children and adolescents with EOP.

## 2. Results

### 2.1. Sample Description

We deeply characterized clinical and sociodemographic features of a total of 23 children and adolescents, including 12 psychiatric patients with psychotic symptoms (EOP group) and 11 psychiatric patients without psychotic symptoms (non-EOP group). We observed that the EOP group was older (mean = 15.00, *p *= 0.030) and had higher years of education (mean = 9.3, *p *= 0.023) (Table 1), nevertheless was close to the Mexican population mean (9.73 years) [27]. Furthermore, patients with EOP had higher hospital psychiatric admissions (*p *= 0.027) and higher prevalence of anxiety and stress disorders (*p *= 0.036). The EOP group had a lower functional score (*p *= 0.00003), and severe GAF score (global assessment of functioning) (*p *= 0.017) (Table 1). Our results shows that EOP had a higher comorbidity and lower functionality.

### 2.2. Epigenetic Age

**Figure 1:**
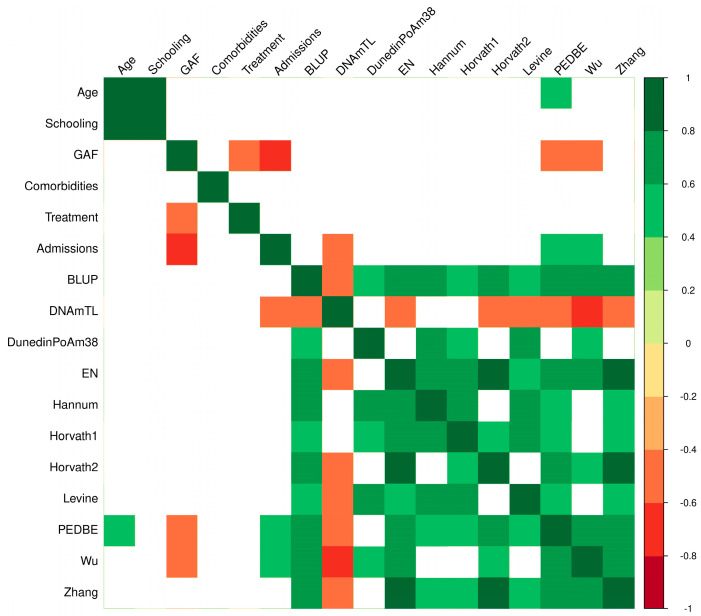
Heatmap of correlations between sociodemographic, clinical characteristics and epigenetic age in psychiatric patients. The heatmap colors correspond to the significance (*p* < 0.05): green indicates a positive correlation, and red indicates a negative correlation. Blank spaces indicate no significant correlation. Best Linear Unbiased Prediction clock (BLUP); DNA methylation-based telomere length (DNAmTL); Dunedin Pace of Aging Methylation (DunedinPoAm38); Elastic Net clock (EN); global assessment of functioning (GAF); Horvath clock (multi-tissue, Horvath-1); Horvath clock (skin and blood, Horvath-2); Levine clock (PhenoAge); Pediatric Buccal Epigenetic clock (PedBE).

There is a hypothesis that patients with psychosis have higher biological age [28]. To explore this, we further characterized 11 epigenetic clocks to evaluate whether if patients with EOP have an increased biological age. Our study found that the epigenetic age was higher in the EOP group compared to the non-EOP group (Table 1). We identified a correlation of epigenetic age with sociodemographic and clinical characteristics with the Wu clock, with a lower functionality measured by the GAF scale being associated with a higher epigenetic age (Figure 1). Moreover, a higher number of admissions was correlated with an increased epigenetic age. Additionally, the same correlations were observed with the PedBE clock, showing a similar direction of effects. In contrast, the DNAmTL clock showed a negative correlation with the number of admissions (Table 2).

In addition, we observed that children and adolescent patients with psychosis were associated with accelerated epigenetic age (Levine clock, β = 5.07, CI 95 = (2.74, 7.40), *p *= 0.001), Furthermore, clinical characteristics appeared to influence this accelerated epigenetic age. Our findings show that a reduction in schooling (Levine clock, β = −5.01, CI 95 = (−7.55, −2.48), *p *= 0.001), a higher number of comorbidities (BLUP clock, β = 0.49, CI 95 = (0.02, 0.97), *p *= 0.041) and more admissions (Wu clock, β = 0.81, CI 95 = (0.03, 1.60), *p *= 0.042) were associated with higher epigenetic age in Mexican patients with EOP (Table 3).

### 2.3. Epigenome-Wide Association Study

**Figure 2:**
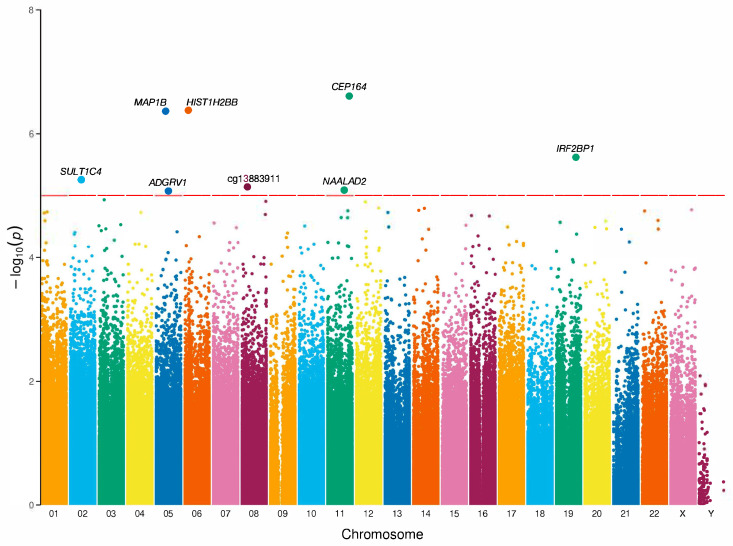
Manhattan plot of EWAS analysis in EOP. Manhattan plot of CpG sites and *p*-values. The *X*-axis represents the chromosome position, and the *Y*-axis represents −log_10_(*p*). The red horizontal line parallel to the *X*-axis denotes nominal significance (*p* < 1 × 10^−5^). All loci with a *p*-value < 1 × 10^−5^ are annotated to genes according to the human genome assembly (hg19). The association models are adjusted for sex, age, and five surrogate variables. Adhesion G Protein-Coupled Receptor V1 (*ADGRV1*); Histone Cluster 1 H2B Family Member B (*HIST1H2BB*); Centrosomal Protein 164 (*CEP164*); Interferon Regulatory Factor 2 Binding Protein 1 (*IRF2BP1*); Microtubule-Associated Protein 1B (*MAP1B*); N-Acetylated α-Linked Acidic Dipeptidase 2 (*NAALAD2*); Sulfotransferase Family 1C Member 4 (*SULT1C4*). λ = 0.9783.

Differential methylation analysis was performed to associate epigenetic risk markers between EOP and non-EOP groups in Mexican patients. We identified eight differentially methylated CpG sites associated with EOP at nominal significance, CpG sites are distributed across six chromosomes (Figure 2). These sites were mapped to seven genes: *ADGRV1* (Adhesion G Protein-Coupled Receptor V1), *HIST1H2BB* (Histone Cluster 1 H2B Family Member B), *CEP164* (Centrosomal Protein 164), *IRF2BP1* (Interferon Regulatory Factor 2 Binding Protein 1), *MAP1B* (Microtubule-Associated Protein 1B), *NAALAD2* (N-Acetylated α-Linked Acidic Dipeptidase 2), and *SULT1C4* (Sulfotransferase Family 1C Member 4). The annotation indicated that four sites were situated in gene bodies, two sites in exons, and one site in a transcription start site (TSS200). In terms of their location relative to CpG islands, 25% of the sites were within the island, 25% on the island shores, and 50% in the open sea. Additionally, six CpG sites had lower methylation values, while two CpG sites showed higher values in the EOP group compared to the non-EOP group (Table 4). In deep research of CpG methylation sites, there are reports of cg06583549, cg08523325, cg20150189, and cg26028573 in the EWAS catalog. Similarly, we conducted an enrichment analysis using the Enrichr tool and found associations between the seven annotated genes and pathways related to aspartate and asparagine metabolism, the cytosolic sulfonation of small molecules, and Schwann cell myelination (Supplementary Sheet S1).

### 2.4. Methylation Risk Score

**Figure 3:**
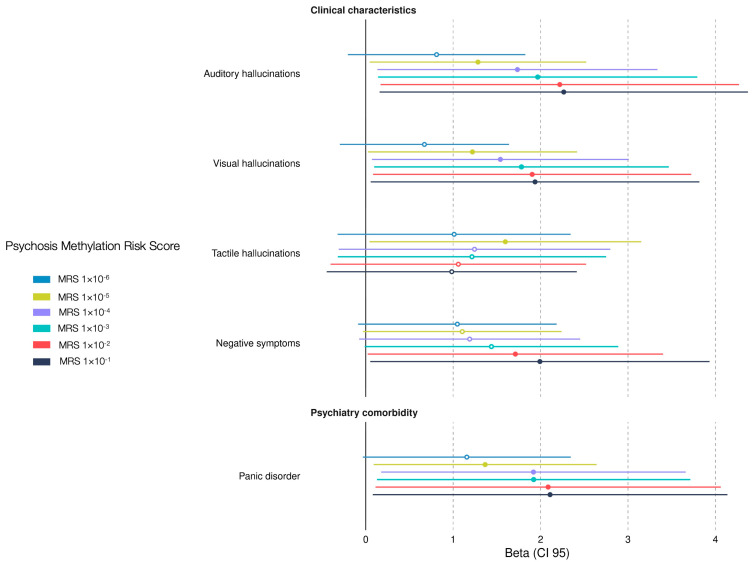
Association between clinical characteristics and early-onset psychosis methylation risk score (EOP-MRS) in psychiatric patients. The β-values represent the estimated coefficient (β) from logistic regression analysis with a 95% confidence interval (CI 95). Significant associations between clinical characteristics and MRS are displayed as solid points (*p* < 0.05), while non-significant results are displayed as hollow points. Each MRS calculated corresponds to a different cutoff *p*-value from the EWAS. The forest plot shows only significant associations among the sixty-eight clinical characteristics.

Epigenetic biomarkers could serve as predicting tools for psychosis [29], and currently there is no epigenetic biomarker for EOP. We constructed the EOP-MRS to associate the biomarker with sociodemographic and clinical characteristic in children and adolescent Mexican patients with EOP. Our findings show that panic disorder, auditory hallucinations, and visual hallucinations were associated with a higher MRS using five different *p*-value thresholds: MRS_1×10^−1^_ (β = 2.10, CI 95 = 0.53–4.70; β = 2.26, CI 95 = 0.63–4.93; β = 1.93, CI 95 = 0.47–4.31; respectively); MRS_1×10^−2^_ (β = 2.08, CI 95 = 0.55–4.64; β = 2.21, CI 95 = 0.63–4.85; β = 1.90, CI 95 = 0.47–4.23; respectively); MRS_1×10^−3^_ (β = 1.92, CI 95 = 0.50–4.23; β = 1.96, CI 95 = 0.54–4.31; β = 1.78, CI 95 = 0.44–3.94; respectively); MRS_1×10^−4^_ (β = 1.91, CI 95 = 0.52–4.16; β = 1.73, CI 95 = 0.45–3.78; β = 1.54, CI 95 = 0.33–3.39; respectively); and MRS_1×10^−5^_ (β = 1.36, CI 95 = 0.28–2.95; β = 1.28, CI 95 = 0.23–2.82; β = 1.22, CI 95 = 0.19–2.69; respectively). Furthermore, a higher MRS was associated with tactile hallucinations (MRS_1×10^−5^_, β = 1.59, CI 95 = 0.25–3.48), and negative symptoms (MRS_1×10^−1^_, β = 1.99, CI 95 = 0.48–4.46; MRS_1×10^−2^_, β = 1.71, CI 95 = 0.37–3.87) (Figure 3). Three out of six thresholds (MRS_1×10^−5^_, MRS_1×10^−2^_, and MRS_1×10^−1^_) predicted three clinical manifestations of psychosis and one psychiatric comorbidity. Our results shows that our EOP-MRS may be an indicator for visual and auditive hallucinations in Mexican children and adolescent patients.

## 3. Discussion

This study performed the first characterization of genome-wide epigenetic biomarkers in Mexican children and adolescents with EOP and further explored the correlations and associations between epigenetic age (11 epigenetic clocks), MRS and sociodemographic and clinical characteristics in Mexican children and adolescents with EOP.

The current study demonstrated that lower levels of functioning, admissions, and anxiety–stress disorder were associated with EOP. Our findings are consistent with a study of European patients with FEP (first episode of psychosis), who had lower GAF scores compared to other psychiatric groups [30]. These results support the hypothesis that a lower global functioning may be influenced by psychiatric comorbidities and admissions, leading to a poor prognosis [31] or premature death [32].

### 3.1. Years of Schooling Was Associated with Epigenetic Age in EOP

The present study shows accelerated epigenetic age in psychiatric patients with EOP. Consistent with our results, previous work identified that monozygotic twins with psychiatric disorders had an accelerated epigenetic age during early adolescence measured with the Wu clock [19], and in Mexican adults, the presence of a mental disorder accelerated Horvath’s epigenetic age in discordant monozygotic twins [33]. In this sense, we suggest that Wu’s clock could be used to estimate epigenetic age in the adolescent Mexican population. Furthermore, the accelerated epigenetic age was associated with fewer years of schooling in EOP, suggesting that more years of schooling could be better to reduce biological age. This association may be influenced by exposure to psychosocial stressors, such as increased academic demands, and unhealthy lifestyles. Additionally, factors characteristic of the Mexican population with low socioeconomic status—such as limited access to health resources and lower self-care literacy—may contribute to allostatic load, potentially activating the hypothalamic–pituitary–adrenal (HPA) axis, altering cortisol release, and modulating dopamine response [9]. This biological response could promote accelerated biological aging through DNA methylation. In accordance with our results, previous work in the Mexican population showed that schooling influences epigenetic age [34]. We consider that schooling could influence biological age in Mexicans with EOP. Notably, the lack of concordance between different epigenetic clocks could reflect how each clock is calibrated to specific sets of CpGs or tissues. Nevertheless, further research is still needed to explore the effect of other stressors and social determinants of health on the relationship between EOP and epigenetic age [35].

### 3.2. EWAS Suggested Potential Novel Associations with EOP

Our EWAS analysis identified eight CpG sites associated with EOP at a nominal level. Nonetheless, a previous meta-analysis reported 95 differentially methylated positions associated with psychosis in adult patients [14]. The *MAP1B* gene regulates axon growth and synaptic plasticity [36], and its dysregulation leads to disruptions in neurogenesis and synaptic plasticity [37]. The *HIST1H2BB* gene is involved in cell motility [38] and neurodevelopment [39]. In addition, methylation changes in the *CEP164* gene are associated with brainstem malformation [40], and the IRF2BP1 gene is involved in immune suppression [41]. There is evidence that *SULT1C4*, *NAALAD2*, and *ADGRV1* participate in neurotransmitter regulation [42], glutamate dysregulation in schizophrenia [43], and epilepsy and audiovisual disorders [44,45]. All these findings indicate that DNA methylation is implicated in neurodevelopmental disorders, immune modulatory pathways, and neurotransmitter regulation in EOP patients. This suggestion is based on the following findings: (i) a reduced level of methylation is observed at cg24772138, cg05100917, cg06583549, cg20150189, cg08523325, and cg27181762; (ii) a higher level of methylation is observed at cg26028573 and cg13883911. This result should be interpretated with caution, as the nominal threshold represents exploratory work. Further studies are needed to replicate our findings.

We identified four CpG sites previously reported in EWASs [46,47,48,49]. Islam et al. (2019) suggest that cg06583549, cg08523325, cg20150189, and cg26028573 present DNAm concordance between buccal epithelial cells and peripheral blood cells in pediatric patients [46]. Furthermore, these sites are associated with children’s neurodevelopment [49] and HIV infection in adults [48]. In addition, cg26028573 was associated with alcohol consumption [47]. Notably, this association has been reported in psychotic patients and individuals with developmental disorders [50]. Our findings support evidence that DNAm signatures overlap across different pediatric tissues in psychotic patients [51]. Moreover, these sites could reflect shifts across the lifespan [52] and neurodevelopmental disorders [53], suggesting that our results could provide insight into children’s neurodevelopment within our population.

### 3.3. Association Between Clinical Characteristics Associated with Psychosis MRS

Our study showed clinical characteristics associated with EOP-MRS. We constructed an EOP-MRS using six different thresholds to obtain varying weighted sums from distinct CpG sites. The MRS_1×10^−5^_ includes the eight CpG sites that were differentially methylated and nominally associated with early-onset psychosis. Our findings demonstrated that increases in this MRS predict three clinical manifestations of psychosis and panic disorder associated with early-onset psychosis. Previous associations of MRS with clinical characteristics have been identified in other populations. For example, in an adult Australian population diagnosed with schizophrenia, the MRS was associated with clozapine administration [54].

The potential utility of our EOP-MRS lies in its ability to predict psychotic symptoms directly measured in the adolescent population. In addition, this score may be determined in individuals who do not exhibit psychosis. Furthermore, to establish it as a biomarker for psychosis, the MRS must be validated in different tissues and evaluated in relation to the stage of the disease. Our study was conducted during the prodromal phase, suggesting its potential utility in identifying risk factors. However, it could be also explored as a biomarker for treatment response, disease severity, or progression in clinical applications. Our EOP-MRS (the most predictive ones: MRS_1×10^−5^_, MRS_1×10^−2^_, and MRS_1×10^−1^_) could represent a predictive tool to identify panic disorder and hallucinations in Mexican children and the adolescent population. We suggest that EOP-MRS reflects the cumulative effect of multiple epigenetic markers associated with EOP and recapitulates its clinical manifestations.

### 3.4. Limitations

We considered several limitations: This is a hypothesis-generating study rather than a conclusive investigation of the biological mechanisms involved in early-onset psychosis. A small sample of psychiatric patients with EOP was included in our research, which limits our statistical power to detect associations and transform this study into an exploratory one. Additionally, epigenetic studies are not ideal for identifying causal risk factors, as DNA methylation (DNAm) may be influenced by environmental confounders. Moreover, EPIC arrays cover only 3% of CpG sites in the genome, and polymorphisms and mutations were not accounted for in psychosis risk calculations. However, this could be addressed using different technologies, such as third-generation sequencing for quantifying methylation levels. Furthermore, DNAm profiles focus on peripheral blood as a surrogate for brain tissue, and variations associated with early-onset psychosis may not represent specific brain tissue biomarkers in the early stages of the disease. Additionally, epigenetic age, EWAS, and psychosis MRS associated with EOP could be biased due to ancestry, pubertal stage, childhood adversity, or environmental exposures not included in the analysis. Likewise, the associations between the EOP-MRS and clinical manifestations could be lost after adjusting the MRS model, possibly due to the use of different thresholds and the small sample size. We did not have a replication cohort given the difficulty in recruiting those with EOP. In addition, we should consider pruning, as variability in epigenetic signals may differ across individuals, populations, and ages, particularly during early stages of life. Finally, future research should address these issues including larger samples, DNAm profiles from other tissues, and social determinants of health to replicate our results.

## 4. Materials and Methods

### 4.1. Sample Population

Thirty-three patients with previously diagnosed psychiatric disorders were recruited from the Dr. Juan N. Navarro Children’s Psychiatric Hospital in Mexico City. A psychiatrist assessed all participants according to the DSM-5 (Diagnostic and Statistical Manual of Mental Disorders, 5th Edition). We included patients aged 10 to 18 years of Mexican descent, with the onset of psychotic symptoms before the age of 18. Exclusion criteria included patients with psychosis secondary to a brain infection, neurodegenerative disease, or severe neurodevelopmental disorder.

### 4.2. Study Design

This was a cross-sectional study with a convenience sample design. Recruitment took place from January 2022 to July 2023. The sample was divided into two groups: the EOP group (patients with psychotic symptoms) and the non-EOP group (patients without psychotic symptoms).

The clinical evaluation of patients was performed using the Kiddie Schedule for Affective Disorders and Schizophrenia Present and Lifetime version DSM-5 (K-SADS PL-5), Spanish edition, validated in children and the adolescent Mexican population [55]. Each individual diagnosis was categorized into one of five groups as follows: (1) mood disorders (disruptive mood dysregulation disorder, dysthymia, major depressive disorder, bipolar disorder); (2) anxiety and stress disorders (separation anxiety disorder, agoraphobia, panic disorder, social phobia, generalized anxiety disorder, post-traumatic stress disorder); (3) conduct disorders (enuresis, encopresis, oppositional defiant disorder, conduct disorder, tic disorder, obsessive-compulsive disorder); (4) neurodevelopmental disorders (attention deficit/hyperactivity disorder, autism spectrum disorder); (5) eating disorders (bulimia nervosa, other eating disorders).

Clinicians collected sociodemographic information of age, gender, schooling (completed years of education), and clinical data, including weight, height, body mass index (BMI), number of psychiatric hospitalizations (admissions), number of medications (psychiatric treatment), and number of psychiatric comorbidities. BMI values were converted into z-scores with standard deviations. The global assessment of functioning (GAF) was measured on a 100-point scale, where lower scores indicated severe symptoms or impairment [56]. GAF is validated in Spanish and adolescent populations [57]. At the end of the interview, we collected a peripheral venous blood sample (4 mL) from each patient.

### 4.3. DNA Extraction

Genomic DNA was obtained from whole blood samples using the DNA kit (Qiagen, Germantown, MD, USA), according to the manufacturer’s instructions. Quality and integrity were evaluated using a NanoDrop spectrophotometer (Thermo Fisher Scientific, Waltham, MA, USA). Sample processing for the methylation study protocol was performed with a genomic DNA concentration of 80 ng/mL. The genomic DNA was bisulfite-converted using the EZ DNA Methylation Kit (Zymo Research Corporation, Irvine, CA, USA), according to the manufacturer’s protocol, “Infinium Assay for Methylation Protocol” (Illumina Inc., San Diego, CA, USA), specific for the “Infinium Methylation EPIC BeadChip Kit.” The Microarray Unit at the National Institute of Genomic Medicine (Mexico City, Mexico) processed the microarray.

### 4.4. Genomic-Wide Quantification of DNA Methylation

Fluorescence intensities were transformed into IDAT files using GenomeStudio software version 2.0 (Illumina, USA). The IDAT data were imported into the R environment. β-normalized methylation levels were obtained following the ENmix (1.22.0) pipeline. Preprocessing included background correction, RELIC dye bias correction, and RCP probe-type bias adjustment. We removed CpG sites with low variability (12,730 CpG sites) and sites that coincided with SNP loci from the analysis. Additionally, 10 samples were eliminated due to sex mismatch, poor quality, and being outliers. Five surrogate variables were estimated to correct for batch effects using ‘csva()’; and cell proportions (neutrophils, monocytes, natural killer cells, CD8+ T-lymphocytes, CD4+ T-lymphocytes, and B-lymphocytes) were imputed using ‘estimateCellProp()’ (ENmix R library). Finally, 848,643 CpG sites from 23 samples were analyzed to obtain the methylation β (β)- and M-values.

### 4.5. Epigenetic Clocks

Epigenetic age was estimated using β-values with two libraries. The methylClock and methylCIPHER packages were chosen for their broad availability of epigenetic calculators. Eleven epigenetic clocks were selected from all three generations of age estimators. Each epigenetic clock was trained on different tissues and different CpG sites to capture aging from diverse sources using different methodological algorithms. The Wu and Pediatric Buccal Epigenetic (PedBE) clocks were included because they are designed to predict age in children and adolescents. The Dunedin Pace of Aging (DunedinPoAm38) and Levine (PhenoAge) clocks were selected for their ability to calculate biological age and predict mortality. DNA methylation-based telomere length (DNAmTL), Hannum, Horvath-1 (multi-tissue), and Zhang clocks were included because they predict chronological age in blood samples. The Best Linear Unbiased Prediction (BLUP), Elastic Net (EN), and Horvath-2 (skin and blood) clocks were chosen because they were trained on blood, skin, and saliva samples using different arrays (450k and EPIC arrays) [58,59,60,61,62,63,64,65,66].

### 4.6. Statistical Analysis

Clinical characteristics are presented as means and standard deviations for continuous variables, and as frequencies and percentages for categorical variables. Comparisons between groups were made using Student’s *t*-test or the Mann–Whitney U test. The associations were assessed with the chi-square test (χ^2^). In psychiatric patients the correlations between epigenetic age and clinical and sociodemographic characteristics were calculated using corrplot R package. Multiple linear regressions (stepwise regression) were performed to associate sociodemographic and clinical characteristics with epigenetic age (the response variable). Statistical analysis was conducted using R software, version 4.3.3 (https://CRAN.R-project.org, accessed 30 December 2024). The significance level was set at *p* < 0.05.

### 4.7. EWAS and Methylation Risk Score

Genome-wide CpG association analysis with EOP was performed using normalized β-values. EOP was the response variable, and each individual CpG methylation β-value served as the predictor of interest. A logistic model was implemented using the ‘CpGassoc()’ R package [67], with PC1 (principal component 1), PC2, PC3, PC4, PC5, age, and sex included as covariates. A variance inflation factor (VIF) was used to detect multicollinearity among predictors and to select covariates. A QQ plot was examined for evidence of genomic inflation (λ = 0.9783, Figure S1). We manually search for previous associations of the CpG sites using the EWAS catalog [68].

CpG sites were stratified by cut-off *p*-values from the EWAS results to calculate the EOP-MRS (methylation risk score to psychosis): MRS_1×10^−6^_ (3 CpG sites, *p* < 1 × 10^−6^), MRS_1×10^−5^_ (8 CpG sites, *p* < 1 × 10^−5^), MRS_1×10^−4^_ (101 CpG sites, *p* < 1 × 10^−4^), MRS_1×10^−3^_ (997 CpG sites, *p* < 1 × 10^−3^), MRS_1×10^−2^_ (9832 CpG sites, *p* < 1 × 10^−2^), MRS_1×10^−1^_ (88,916 CpG sites, *p* < 1 × 10^−1^).

(1) $$MRS=\sum_{n=CpG}\left(M\;value\;\times \;Z\;value\right)$$

The MRS was calculated as the sum of the individual products of the M-values and the Z-values (effect size/standard error) for each CpG site (CpG1 + CpG2 + … + CpGn). The resulting score was then normalized into z-scores.

(2) $$MRS\;z\;score=\frac{MRS\;score-mean\;MRS\;value}{standard\;deviation}$$

Finally, linear and logistic regressions were performed to assess associations between EOP-MRS and sociodemographic and clinical characteristics. Sociodemographic and clinical variables served as the response variable, and the EOP-MRS was considered a predictor of interest.

## 5. Conclusions

We found that patients with EOP have lower levels of global functioning and accelerated epigenetic age. Furthermore, fewer years of education, comorbidity, and psychiatric hospital admissions impact epigenetic age. Additionally, our study suggests associations between DNAm and EOP, specifically in genes involved in immune modulatory and neurotransmission pathways. Finally, we developed an EOP-MRS that could predict clinical manifestations of psychosis and psychiatric comorbidity associated with EOP. This score could be used as a risk biomarker for EOP in Mexican adolescents.

## Figures and Tables

**Table 1:** Sociodemographic and clinical characteristics of psychiatric patients.

Characteristic		EOP (*n *= 12)	Non-EOP (*n *= 11)	*p*
Age	years ± SD	15.5 ± 1.56	13.36 ± 2.57	**0.030 ^a^**
Gender	Male, *n* (%)	6 (50)	7 (64)	0.680 ^c^
	Female, *n* (%)	6 (50)	4 (36)	
Education	years ± SD	9.3 ± 1.96	7.2 ± 2.05	**0.023 ^a^**
Body mass index	z-score, mean ± SD	1.08 ± 1.15	0.67 ± 1.50	0.368 ^a^
Psychiatric admissions	*n* (%)	7 (58)	1 (9)	**0.027 ^c^**
	Total, median (min–max)	1 (0–4)	0 (0–1)	**0.016 ^b^**
Psychiatric comorbidity	*n* (%)	12(100)	9 (81)	0.370 ^c^
	Mood disorders, *n* (%)	11 (91)	8 (72)	0.316 ^c^
	Anxiety and stress disorders, *n* (%)	10 (83)	4 (36)	**0.036 ^c^**
	Conduct disorders, *n* (%)	5 (41)	5 (45)	1 ^c^
	Neurodevelopment disorders, *n *(%)	1 (8)	4 (36)	0.155 ^c^
	Eating disorder, *n* (%)	4 (33)	0	0.093 ^c^
GAF	Total score, mean ± SD	43.33 ± 15.14	74 ± 6.41	**0.00003 ^a^**
	Minimal, *n* (%)	1 (8)	3 (27)	**0.017 ^c^**
	Mild, *n* (%)	1 (8)	2 (18)	
	Moderate, *n* (%)	2 (16)	0	
	Severe, *n* (%)	8 (66)	0	
Epigenetic age	Wu’s clock, mean ± SD	11.08 ± 0.89	10.29 ± 0.78	**0.015**

Abbreviations: BMI = body mass index; GAF = global assessment of functioning; SD = standard deviation; T = Student’s *t*-test; U = Mann–Whitney U test; χ^2^ = chi-square test; ^a^ = statistic from Student’s *t*-test; ^b^ = statistic from Mann–Whitney–Wilcoxon test; ^c^ = statistic from χ^2^ test. Notes: Fisher’s exact test was applied when values <5. Bold values denote statistical significance, *p* < 0.05.

**Table 2:** Correlations between sociodemographic, clinical characteristics and epigenetic age in psychiatric patients.

Epigenetic Calculator	Age r, *p*	GAF r, *p*	Admissions r, *p*
BLUP	0.37, 0.079	−0.40, 0.056	0.40, 0.056
DNAmTL	−0.12, 0.555	0.27, 0.198	**−0.43, 0.038**
DunnedinPoAm38	0.26, 0.224	−0.17, 0.434	0.19, 0.368
EN	0.26, 0.220	−0.26, 0.217	0.40, 0.057
Hannum	0.25, 0.238	−0.23, 0.274	0.29, 0.174
Horvath-1	0.18, 0.398	0.13, 0.553	−0.07, 0.742
Horvath-2	0.03, 0.848	−0.17, 0.427	0.34, 0.107
Levine	0.35, 0.100	−0.05, 0.806	0.09, 0.672
PedBE	**0.41, 0.046**	**−0.53, 0.008**	**0.56, 0.005**
Wu	0.30, 0.150	**−0.45, 0.027**	**0.49, 0.015**
Zhang	0.24, 0.253	−0.21, 0.324	0.35, 0.097

Abbreviations: R = Pearson’s correlation, Best Linear Unbiased Prediction clock (BLUP); DNA methylation-based telomere length (DNAmTL); Dunedin Pace of Aging Methylation (DunedinPoAm38); Elastic Net clock (EN); global assessment of functioning (GAF); Horvath’s clock (multi-tissue, Horvath-1); Horvath’s clock (skin and blood, Horvath-2); Levine’s clock (PhenoAge); Pediatric Buccal Epigenetic clock (PedBE). Notes: Correlations were made with either Pearson or Spearman. Bold indicates *p* < 0.05.

**Table 3:** Summary of the stepwise regression between epigenetic clocks and sociodemographic and clinical characteristics in psychiatric patients.

Epiclock	Age (Years)		Sex		Schooling (Years)		Comorbidity		Admissions	
	β, SE (CI 95)	*p*	β, SE (CI 95)	*p*	β, SE (CI 95)	*p*	β, SE (CI 95)	*p*	β, SE (CI 95)	*p*
BLUP	1.78, 0.55 (0.62, 2.94)	**0.004**	-	-	−1.82, 0.54 (−2.96, −0.67)	**0.003**	0.49, 0.22 (0.02, 0.97)	**0.041**	-	-
DNAmTL	−0.07, 0.02 (−0.11, −0.02)	**0.006**	-	-	0.08, 0.02 (0.03, 0.13)	**0.001**	-	**-**	−0.13, 0.05 (−0.24, −0.02)	**0.017**
EN	1.36, 0.58 (0.12, 2.60)	**0.032**	-	-	−1.47, 0.58 (−2.69, −0.25)	**0.020**	-	-	-	-
Horvath-1	1.75, 0.70 (0.26, 3.24)	**0.023**	-	-	-	-	-	-	-	-
Horvath-2	0.60, 0.26 (0.04, 1.16)	**0.034**	-	-	−0.90, 0.26 (−1.45, −0.35)	**0.002**	0.31, 0.10 (0.08, 2.38)	**0.010**	-	-
Levine	5.07, 1.10 (2.74, 7.40)	**0.001**	8.41, 2.37 (3.4, 13.40)	**0.002**	−5.01, 1.20 (−7.55, −2.48)	**0.001**	-	-	-	-
PedBE	0.35, 0.10 (0.13, 0.58)	**0.003**	-	-	−0.31, 0.10 (−0.53, −0.09)	**0.008**	-	-	-	-
Wu	-	-	-	-	-	-	-	-	0.81, 0.37 (0.03, 1.60)	**0.042**
Zhang	1.93, 0.66 (0.56, 3.31)	**0.008**	-	-	−1.81, 0.69 (−3.25, −0.36)	**0.016**	-	-	-	-

Abbreviations: β = estimated coefficient; SE = standard error; T *= t*-value; BLUP = Best Linear Unbiased Prediction clock; DNAmTL = DNA methylation-based telomere length; EN = Elastic Net clock; Horvath-1 = Horvath clock (multi-tissue); Horvath-2 = Horvath clock (skin and blood); PedBE = Pediatric Buccal Epigenetic clock. Bold values denote statistical significance at the *p* < 0.05 level. Comorbidity values were represented as the number of psychiatric disorders; sex was denoted as a factor (0 = male, 1 = female); admissions were coded as a factor (0 = no, 1 = yes).

**Table 4:** Top differentially methylated positions in EOP.

CpG	Gene Annotation	Chr	Position	Relation to Island	β	SE	T	*p*
cg24772138	*MAP1B* (Body)	5	71,405,539	S Shore	−0.4483	0.0508	−8.8233	4.30 × 10^−7^
cg26028573	*HIST1H2BB* (Exon)	6	26,043,587	N Shore	0.2340	0.0264	8.8374	4.22 × 10^−7^
cg05100917	*CEP164* (TSS200)	11	117,198,460	Island	−0.5790	0.0627	−9.2343	2.48 × 10^−7^
cg20150189	*SULT1C4* (Body)	2	108,999,219	Open Sea	−0.3525	0.0498	−7.0718	5.58 × 10^−6^
cg27181762	*ADGRV1* (Body)	5	90,195,345	Open Sea	−0.5829	0.0860	−6.7701	9.02 × 10^−6^
cg13883911	-	8	33,430,120	Open Sea	0.3104	0.0449	6.9051	7.26 × 10^−6^
cg08523325	*NAALAD2* (Body)	11	89,901,450	Open Sea	−0.3064	0.0448	−6.8337	8.14 × 10^−6^
cg06583549	*IRF2BP1* (Exon)	19	46,387,962	Island	−0.2669	0.0350	−7.6212	2.40 × 10^−6^

Abbreviations: Chr = chromosome; β = effect size; TSS = transcription start site; T *= t*-statistic; SE = standard error. Adhesion G Protein-Coupled receptor V1 (*ADGRV1*); Histone Cluster 1 H2B Family Member B (*HIST1H2BB*); Centrosomal Protein 164 (*CEP164*); Interferon Regulatory Factor 2 Binding Protein 1 (*IRF2BP1*); Microtubule-Associated Protein 1B (*MAP1B*); N-Acetylated α-Linked Acidic Dipeptidase 2 (*NAALAD2*); Sulfotransferase Family 1C Member 4 (*SULT1C4*). CpG sites ordered by statistical *p*-value from EWAS analysis in early-onset psychosis.

## Data Availability

The original contributions presented in the study are included in the article/Supplementary Materials, further inquiries can be directed to the corresponding author.

## Supplementary Materials

The following supporting information can be downloaded at: https://www.mdpi.com/article/10.3390/genes16050591/s1, Figure S1: QQ plot of EWAS of early-onset psychosis; Table S1: Clinical characteristics of early-onset psychosis; Sheet S1: Sociodemographic and clinical associations with EOP-MRS, and EWAS catalog; Figure S2: Most frequent psychiatric comorbidities; Table S2: Summary of the stepwise regression between Best Linear Unbiased Prediction’s clock (BLUP) and sociodemographic and clinical characteristics: Figure S3: β-value distribution (A) before and (B) after quality control; Table S3: Summary of the stepwise regression between DNA methylation-based telomere length (DNAmTL) and sociodemographic and clinical characteristics; Figure S4: Distribution of T-statistic values of EWAS; Table S4: Summary of the stepwise regression between Dunedin Pace of Aging Methylation (DunedinPoAm38) and sociodemographic and clinical characteristics; Table S5: Summary of the stepwise regression between Elastic Net’s clock (EN) and sociodemographic and clinical characteristics; Table S6: Summary of the stepwise regression between Hannum’s clock and sociodemographic and clinical characteristics; Table S7: Summary of the stepwise regression between Horvath’s clock (multi-tissue) and sociodemographic and clinical characteristics; Table S8: Summary of the stepwise regression between Horvath’s clock (skin and blood) and sociodemographic and clinical characteristics; Table S9: Summary of the stepwise regression between Levine’s clock (PhenoAge) and sociodemographic and clinical characteristics; Table S10: Summary of the stepwise regression between Pediatric Buccal Epigenetic clock (PedBE) and sociodemographic and clinical characteristics; Table S11: Summary of the stepwise regression between Wu’s clock and sociodemographic and clinical characteristics; Table S12: Summary of the stepwise regression between Zhang’s clock and sociodemographic and clinical characteristics; Table S13: Correlations between Best Linear Unbiased Prediction’s clock (BLUP) and sociodemographic and clinical characteristics; Table S14: Correlations between DNA methylation-based telomere length (DNAmTL) and sociodemographic and clinical characteristics; Table S15: Correlations between Dunedin Pace of Aging Methylation (DunedinPoAm38) and sociodemographic and clinical characteristics; Table S16: Correlations between Elastic Net clock (EN) and sociodemographic and clinical characteristics; Table S17: Correlations between Hannum’s clock and sociodemographic and clinical characteristics; Table S18: Correlations between Horvath’s clock (multi-tissue) and sociodemographic and clinical characteristics; Table S19: Correlations between Horvath’s clock (skin and blood) and sociodemographic and clinical characteristics; Table S20: Correlations between Levine’s clock (PhenoAge) and sociodemographic and clinical characteristics; Table S21: Correlations between Pediatric Buccal Epigenetic clock (PedBE) and sociodemographic and clinical characteristics; Table S22: Correlations between Wu clock and sociodemographic and clinical characteristics; Table S23: Correlations between Zhang’s clock and sociodemographic and clinical characteristics; Sheet S1: Sociodemographic and clinical association with EOP-MRS, EWAS catalog, and pathway enrichment analysis [69].

## Abbreviations

The following abbreviations are used in this manuscript:

*ADGRV1*	Adhesion G Protein-Coupled Receptor V1 gene
BLUP	Best Linear Unbiased Prediction
*CEP164*	Centrosomal Protein 164 gene
CpG	Cytosine–guanine site
DNA	Deoxyribonucleic acid
DNAm	Deoxyribonucleic acid methylation
DNAmTL	DNA methylation-based telomere length
DSM-5	Diagnostic and Statistical Manual of Mental Disorders, 5th Edition
DunedinPoAm38	Dunedin Pace of Aging
EN	Elastic Net
EOP	Early-onset psychosis
EOP-MRS	Psychosis methylation risk score
EWAS	Epigenome-wide association study
FEP	First episode of psychosis
GAF	Global assessment of functioning
*HIST1H2BB*	Histone Cluster 1 H2B Family Member B gene
*IRF2BP1*	Interferon Regulatory Factor 2 Binding Protein 1 gene
K-SADS PL-5	Kiddie Schedule for Affective Disorders and Schizophrenia Present and Lifetime version DSM-5
*MAP1B*	Microtubule-Associated Protein 1B gene
MRS	Methylation risk score
*NAALAD2*	N-Acetylated α-Linked Acidic Dipeptidase 2 gene
PC	Principal component
PedBE	Pediatric Buccal Epigenetic
*SULT1C4*	Sulfotransferase Family 1C Member 4 gene
TSS	Transcription start site
VIF	Variance inflation factor
