# Supplementary material for: Characterizing the Social Epigenome in Mexican Patients with Early-Onset Psychosis
Source: Genes (Basel). 2025 May 17;16(5):591. doi: 10.3390/genes16050591 (PMC12111507; doi:10.3390/genes16050591)
Supplement: Supplementary file 1 [file genes-16-00591-s001.zip › Suplemetary figures and tables.pdf]

Supplementary figures and tables.

Table S1. Clinical characteristics of early−onset psychosis.

Psychotic symptom	n (%)
Delusions of reference	9 (75.0)
Delusions of persecutions	4 (33.3)
Grandiose delusions	3 (25.0)
Somatic delusional ideas	4 (33.3)
Other delusional ideas	3 (25.0)
Auditory hallucinations	11 (91.6)
Visual hallucinations	10 (83.3)
Tactile hallucinations	4 (33.3)
Other hallucinations	3 (25.0)
Catatonic behavior	1 (8.3)
Disorganized behavior	4 (33.3)
Innapropiate affection	2 (16.6)
Disorganized speech	2 (16.6)
Negative symptons	9 (75.0)

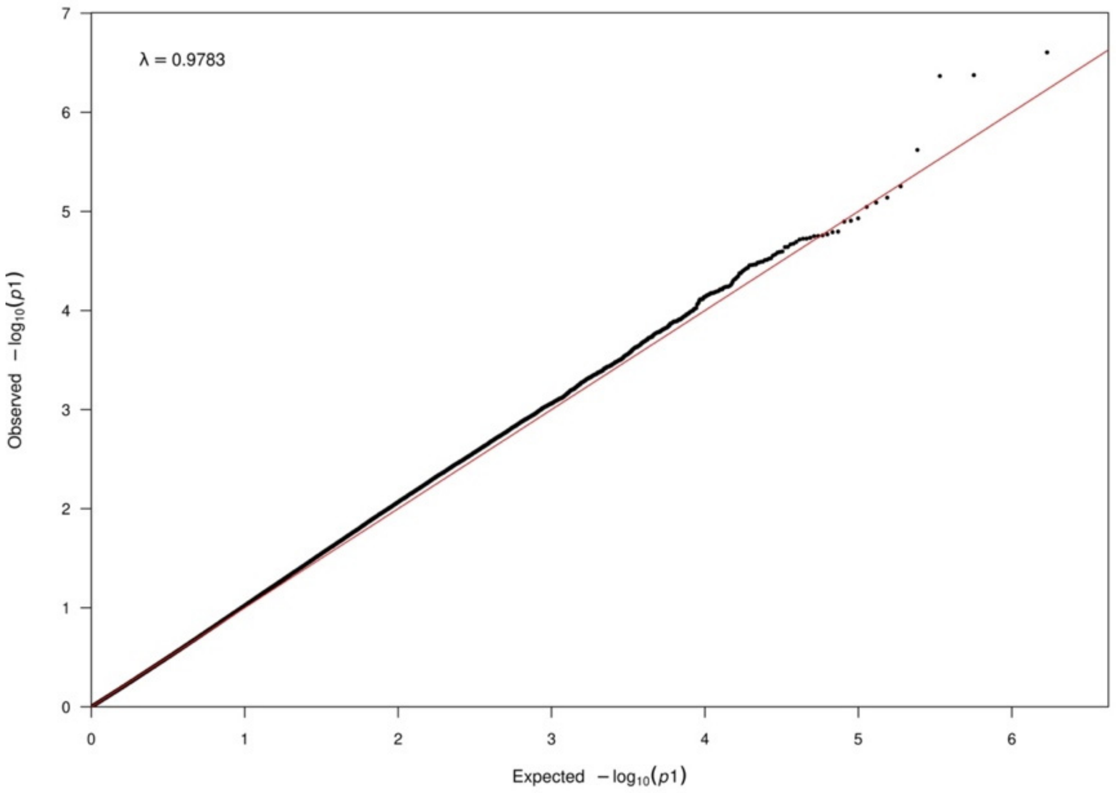

Figure S1. QQ plot of EWAS of early onset psychosis.

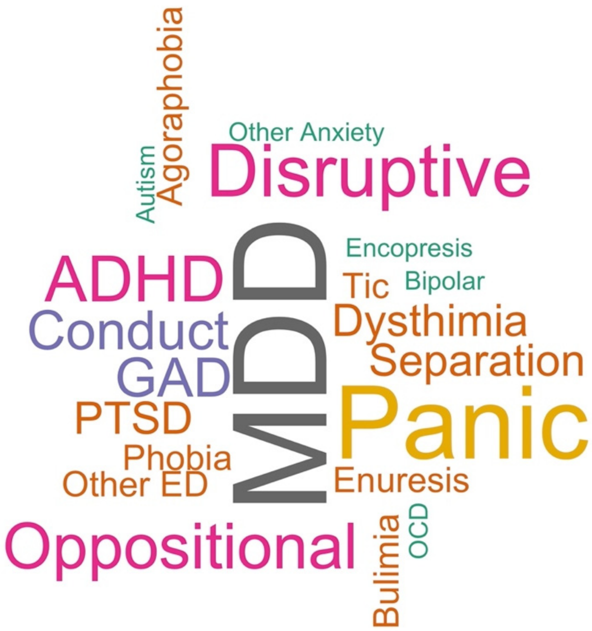

Figure S2. Most frequents psychiatric comorbidities.

ADHD = attention-deficit/hyperactivity disorder; Autism = autism spectrum disorder; Bipolar = bipolar disorder type 1; Conduct = conduct disorder; Disruptive = Disruptive mood dysregulation disorder; GAD = generalized anxiety disorder; MDD = major depressive disorder; OCD = obsessive compulsive disorder; Oppositional = oppositional defiant disorder; Other Anxiety = other specified anxiety disorder; Other ED = other specified eating disorder; Panic = panic disorder; Phobia = social phobia; PTSD = post−traumatic stress disorder; Separation = separation anxiety disorder.

A)

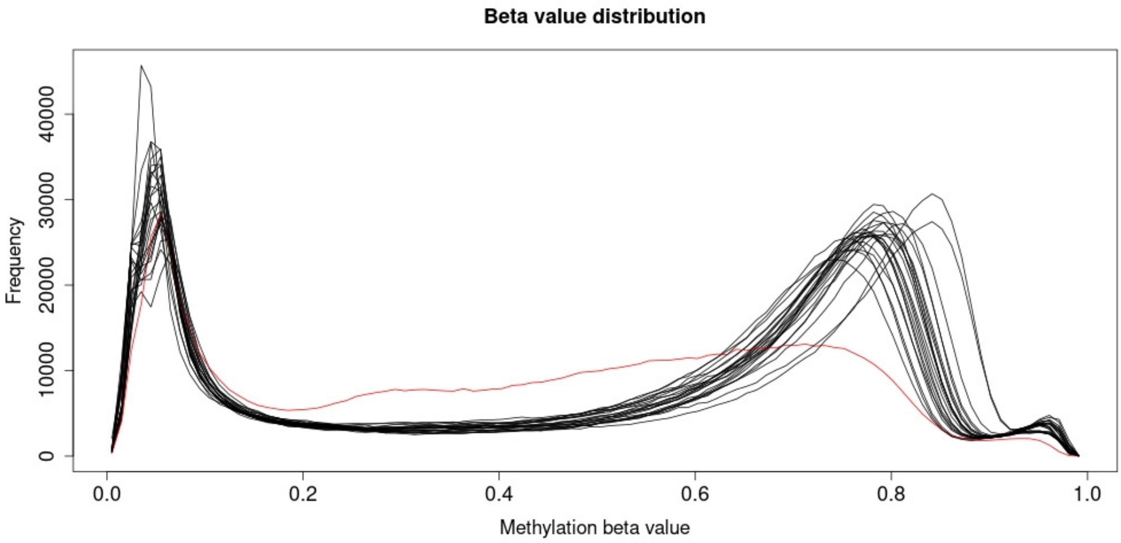

B)

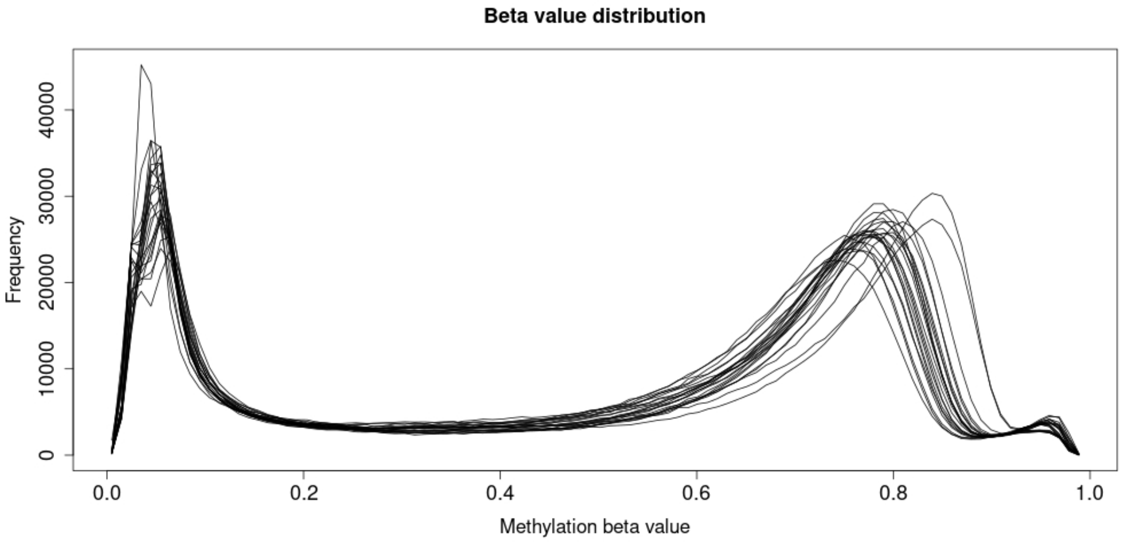

Figure S3. Beta value distribution A) before and B) after quality control.

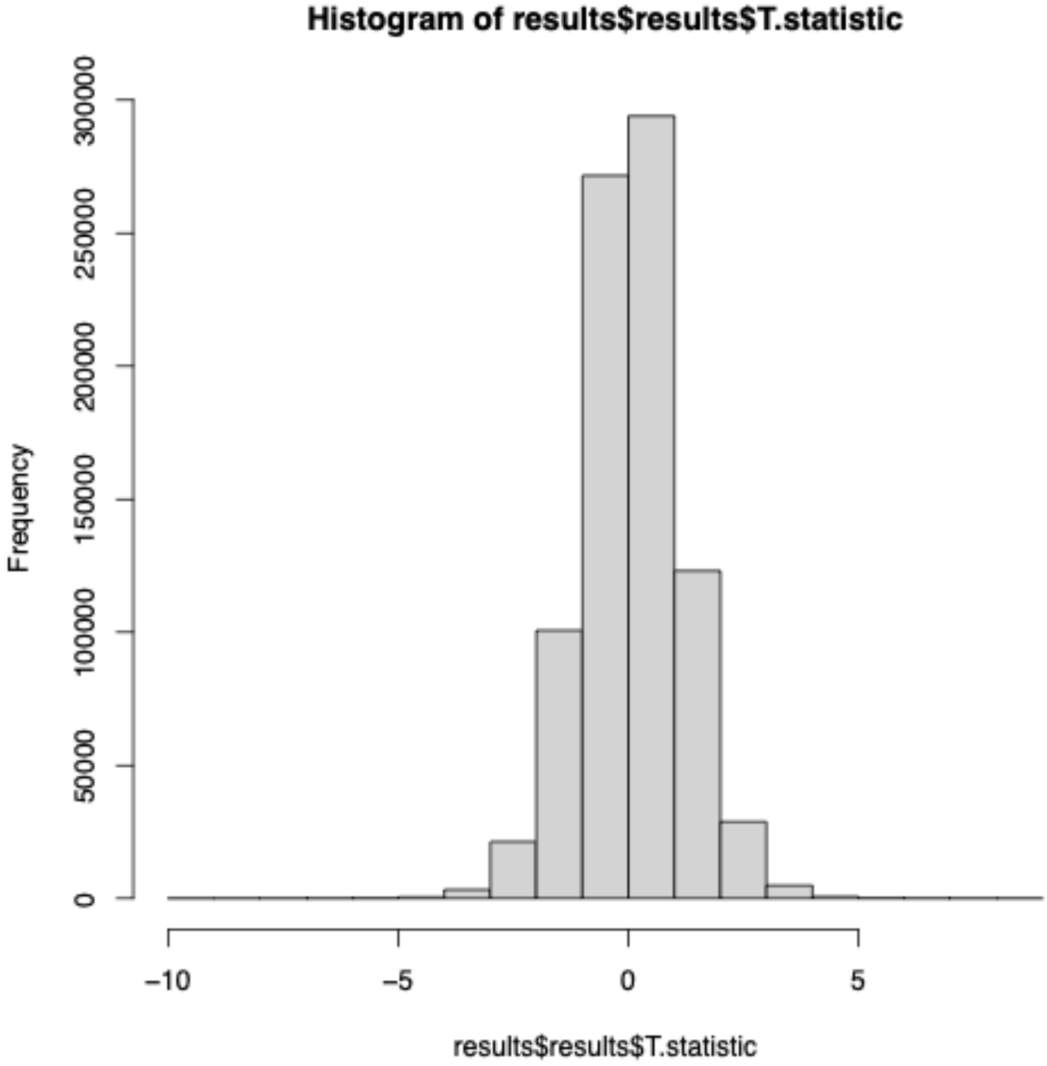

Figure S4. Distribution of T statistic values of EWAS.

Table S2. Summary of the stepwise regression between Best Linear Unbiased Prediction`s clock (BLUP), sociodemographic and clinical characteristics.

Component	Estimate	Standard Error	*t* value	Pr (>|t|)
(Intercept)	-3.3632	4.2495	-0.791	0.43899
Age (years)	1.7868	0.5513	3.241	**0.00454**
Sex	-	-	-	-
Schooling (years)	-1.8203	0.5445	-3.343	**0.00362**
GAF	-	-	-	-
Comorbidity	0.4961	0.2259	2.196	**0.04147**
Treatment	-	-	-	-
Psychiatric admissions	2.3395	1.2254	1.909	0.07232
Residual standard error: 2.471 on 18 degrees of freedom				
Multiple R-squared: 0.6183, Adjusted R-squared: 0.5335				
F-statistic: 7.289 on 4 and 18 degrees of freedom, p-value: **0.001129**				

Abbreviations: GAF = Global Assessment of Function. Bold values denote statistical significance, $p < 0.05$.

Table S3. Summary of the stepwise regression between DNA methylation−based telomere length (DNAmTL), sociodemographic and clinical characteristics.

Component	Estimate	Standard Error	*t* value	Pr (>|t|)
(Intercept)	-7.82097	0.17872	-43.762	**< 2e-16**
Age (years)	-0.07121	0.02310	-3.083	**0.00612**
Sex	-	-	-	-
Schooling (years)	0.08684	0.02288	3.795	**0.00122**
GAF	-	-	-	-
Comorbidity	-	-	-	-
Treatment	-	-	-	-
Psychiatric admissions	-0.13424	0.05147	-2.608	**0.01728**
Residual standard error: 0.1039 on 19 degrees of freedom				
Multiple R-squared: 0.6022, Adjusted R-squared: 0.5394				
F-statistic: 9.588 on 3 and 19 degrees of freedom, p-value: **0.0004546**				

Abbreviations: GAF = Global Assessment of Function. Bold values denote statistical significance, $p < 0.05$.

Table S4. Summary of the stepwise regression between Dunedin Pace of Aging Methylation (DunedinPoAm38), sociodemographic and clinical characteristics.

Component	Estimate	Standard Error	*t* value	Pr (>|t|)
(Intercept)	1.05312	0.01375	76.59	**<2e-16**
Age (years)	0.03917	0.02331	1.68	0.108
Sex	-	-	-	-
Schooling (years)	-	-	-	-
GAF	-	-	-	-
Comorbidity	-	-	-	-
Treatment	-	-	-	-
Psychiatric admissions	-	-	-	-
Residual standard error: 0.05325 on 21 degrees of freedom				
Multiple R-squared: 0.1185, Adjusted R-squared: 0.07653				
F-statistic: 2.823 on 1 and 21 degrees of freedom, p-value: 0.1077				

Abbreviations: GAF = Global Assessment of Function. Bold values denote statistical significance, $p < 0.05$.

Table S5. Summary of the stepwise regression between Elastic Net`s clock (EN), sociodemographic and clinical characteristics.

Component	Estimate	Standard Error	*t* value	Pr (>|t|)
(Intercept)	4.4888	4.5461	0.987	0.3365
Age (years)	1.3653	0.5898	2.315	**0.0326**
Sex	-	-	-	-
Schooling (years)	-1.4756	0.5825	-2.533	**0.0208**
GAF	-	-	-	-
Comorbidity	0.3323	0.2417	1.375	0.1861
Treatment	-	-	-	-
Psychiatric admissions	1.9003	1.3110	1.450	0.1644
Residual standard error: 2.643 on 18 degrees of freedom				
Multiple R-squared: 0.4469, Adjusted R-squared: 0.324				
F-statistic: 3.637 on 4 and 18 degrees of freedom, p-value: **0.02431**				

Abbreviations: GAF = Global Assessment of Function. Bold values denote statistical significance, $p < 0.05$.

Table S6. Summary of the stepwise regression between Hannum´s clock, sociodemographic and clinical characteristics.

Component	Estimate	Standard Error	*t* value	Pr (>|t|)
(Intercept)	-5.2930	5.8624	-0.903	0.3773
Age (years)	1.5496	0.7510	2.063	0.0523
Sex	-	-	-	-
Schooling (years)	-1.3163	0.7864	-1.674	0.1097
GAF	-	-	-	-
Comorbidity	-	-	-	-
Treatment	-	-	-	-
Psychiatric admissions	-	-	-	-
Residual standard error: 3.691 on 20 degrees of freedom				
Multiple R-squared: 0.1803, Adjusted R-squared: 0.09837				
F-statistic: 2.2 on 2 and 20 degrees of freedom, p-value: 0.1369				

Abbreviations: GAF = Global Assessment of Function. Bold values denote statistical significance, $p < 0.05$.

Table S7. Summary of the stepwise regression between Horvath´s clock (Multi−Tissue), sociodemographic and clinical characteristics.

Component	Estimate	Standard Error	*t* value	Pr (>|t|)
(Intercept)	-6.09717	7.05901	-0.864	0.3998
Age (years)	1.75161	0.70634	2.480	**0.0239**
Sex	2.28612	1.54836	1.476	0.1581
Schooling (years)	-1.58935	0.76008	-2.091	0.0519
GAF	0.05531	0.03934	1.406	0.1778
Comorbidity	-	-	-	-
Treatment	3.17692	2.24811	1.413	0.1757
Psychiatric admissions	-	-	-	-
Residual standard error: 2.727 on 17 degrees of freedom				
Multiple R-squared: 0.3361, Adjusted R-squared: 0.1408				
F-statistic: 1.721 on 5 and 17 degrees of freedom, p-value: 0.1836				

Abbreviations: GAF = Global Assessment of Function. Bold values denote statistical significance, $p < 0.05$.

Table S8. Summary of the stepwise regression between Horvath´s clock (Skin & Blood), sociodemographic and clinical characteristics.

Component	Estimate	Standard Error	*t* value	Pr (>|t|)
(Intercept)	5.1329	2.0416	2.514	**0.02166**
Age (years)	0.6045	0.2649	2.282	**0.03486**
Sex	-	-	-	-
Schooling (years)	-0.9031	0.2616	-3.452	**0.00284**
GAF	-	-	-	-
Comorbidity	0.3108	0.1085	2.864	**0.01033**
Treatment	-	-	-	-
Psychiatric admissions	1.1497	0.5887	1.953	0.06657

Residual standard error: 1.187 on 18 degrees of freedom
Multiple R-squared: 0.5971, Adjusted R-squared: 0.5075
F-statistic: 6.668 on 4 and 18 degrees of freedom, p-value: **0.001785**

Abbreviations: GAF = Global Assessment of Function. Bold values denote statistical significance, $p < 0.05$.

Table S9. Summary of the stepwise regression between Levine´s clock "PhenoAge", sociodemographic and clinical characteristics.

Component	Estimate	Standard Error	*t* value	Pr (>|t|)
(Intercept)	-36.3506	8.1268	-4.473	**0.000294**
Age (years)	5.0781	1.1094	4.577	**0.000234**
Sex	8.4138	2.3771	3.540	**0.002342**
Schooling (years)	-5.0176	1.2058	-4.161	**0.000587**
GAF	-	-	-	-
Comorbidity	0.5685	0.3943	1.442	0.166573
Treatment	-	-	-	-
Psychiatric admissions	-	-	-	-

Residual standard error: 4.315 on 18 degrees of freedom
Multiple R-squared: 0.5833, Adjusted R-squared: 0.4908
F-statistic: 6.3 on 4 and 18 degrees of freedom, p-value: **0.002365**

Abbreviations: GAF = Global Assessment of Function. Bold values denote statistical significance, $p < 0.05$.

Table S10. Summary of the stepwise regression between Pediatric Buccal Epigenetic´s clock (PedBE), sociodemographic and clinical characteristics.

Component	Estimate	Standard Error	*t* value	Pr (>|t|)
(Intercept)	3.3961	0.9239	3.676	**0.00173**
Age (years)	0.3565	0.1077	3.309	**0.00390**
Sex	-	-	-	-
Schooling (years)	-0.3144	0.1065	-2.952	**0.00854**
GAF	-	-	-	-
Comorbidity	-	-	-	-
Treatment	0.5649	0.3696	1.528	0.14378
Psychiatric admissions	0.4576	0.2471	1.852	0.08054
Residual standard error: 0.4837 on 18 degrees of freedom				
Multiple R-squared: 0.6163, Adjusted R-squared:0.5311				
F-statistic: 7.229 on 4 and 18 degrees of freedom, p-value: **0.00118**				

Abbreviations: GAF = Global Assessment of Function. Bold values denote statistical significance, $p < 0.05$.

Table S11. Summary of the stepwise regression between Wu´s clock, sociodemographic and clinical characteristics.

Component	Estimate	Standard Error	*t* value	Pr (>|t|)
(Intercept)	8.5031	1.3021	6.530	**2.97e-06**
Age (years)	0.2999	0.1683	1.782	0.0907
Sex	-	-	-	-
Schooling (years)	- 0.2899	0.1667	-1.739	0.0981
GAF	-	-	-	-
Comorbidity	-	-	-	-
Treatment	-	-	-	-
Psychiatric admissions	0.8169	0.3750	2.178	**0.0422**
Residual standard error: 0.7572 on 19 degrees of freedom				
Multiple R-squared: 0.4143, Adjusted R-squared: 0.3218				
F-statistic: 4.479 on 3 and 19 degrees of freedom, p-value: **0.01537**				

Abbreviations: GAF = Global Assessment of Function. Bold values denote statistical significance, $p < 0.05$.

Table S12. Summary of the stepwise regression between Zhang`s clock, sociodemographic and clinical characteristics.

Component	Estimate	Standard Error	*t* value	Pr (>|t|)
(Intercept)	-8.9351	5.1547	-1.733	0.09842
Age (years)	1.9380	0.6603	2.935	**0.00819**
Sex	-1.8101	0.6914	-2.618	**0.01647**
Schooling (years)	-	-	-	-
GAF	-	-	-	-
Comorbidity	-	-	-	-
Treatment	-	-	-	-
Psychiatric admissions	-	-	-	-
Residual standard error: 3.245 on 20 degrees of freedom				
Multiple R-squared: 0.301, Adjusted R-squared: 0.2311				
F-statistic: 4.307 on 2 and 20 degrees of freedom, p-value: **0.02784**				

Abbreviations: GAF = Global Assessment of Function. Bold values denote statistical significance, $p < 0.05$.

Table S13. Correlations between Best Linear Unbiased Prediction`s clock (BLUP), sociodemographic and clinical characteristics.

Variable	R / rho	p value
Age	0.3426	0.1095
Schooling	0.0810	0.7132
GAF	-0.4205	**0.0456**
Comorbidity	0.4354	**0.0378**
Treatment	0.2620	0.2271
Psychiatric admissions	0.4798	**0.0205**

Abbreviations: GAF = Global Assessment of Function. Bold values denote statistical significance, $p < 0.05$.

Table S14. Correlations between DNA methylation−based telomere length (DNAmTL), sociodemographic and clinical characteristics.

Variable	R / rho	p value
Age	-0.1297	0.5552
Schooling	0.1830	0.4032
GAF	0.2780	0.1989
Comorbidity	0.0302	0.8912
Treatment	0.0386	0.861
Psychiatric admissions	-0.5674	**0.0047**

Abbreviations: GAF = Global Assessment of Function. Bold values denote statistical significance, $p < 0.05$.

Table S15. Correlations between Dunedin Pace of Aging Methylation (DunedinPoAm38), sociodemographic and clinical characteristics.

Variable	R / rho	p value
Age	0.2636	0.2242
Schooling	0.2276	0.2961
GAF	-0.1712	0.4346
Comorbidity	0.0154	0.9441
Treatment	0.1868	0.3932
Psychiatric admissions	0.3293	0.1249

Abbreviations: GAF = Global Assessment of Function. Bold values denote statistical significance, $p < 0.05$.

Table S16. Correlations between Elastic Net`s clock (EN), sociodemographic and clinical characteristics.

Variable	R / rho	p value
Age	0.2609	0.2291
Schooling	0.0480	0.8278
GAF	-0.2058	0.3461
Comorbidity	0.2683	0.2157
Treatment	0.0307	0.8894
Psychiatric admissions	0.5426	**0.0074**

Abbreviations: GAF = Global Assessment of Function. Bold values denote statistical significance, $p < 0.05$.

Table S17. Correlations between Hannum´s clock, sociodemographic and clinical characteristics.

Variable	R / rho	p value
Age	0.2559	0.2385
Schooling	0.0764	0.7288
GAF	-0.2376	0.2749
Comorbidity	0.1991	0.3623
Treatment	0.1646	0.4528
Psychiatric admissions	0.2772	0.2003

Abbreviations: GAF = Global Assessment of Function. Bold values denote statistical significance, $p < 0.05$.

Table S18. Correlations between Horvath´s clock (Multi−Tissue), sociodemographic and clinical characteristics.

Variable	R / rho	p value
Age	0.1849	0.3981
Schooling	0.0567	0.7971
GAF	0.1302	0.5536
Comorbidity	0.1667	0.4470
Treatment	0.0127	0.9541
Psychiatric admissions	-0.0402	0.8552

Abbreviations: GAF = Global Assessment of Function. Bold values denote statistical significance, $p < 0.05$.

Table S19. Correlations between Horvath´s clock (Skin & Blood), sociodemographic and clinical characteristics.

Variable	R / rho	p value
Age	0.0321	0.8842
Schooling	-0.2331	0.2844
GAF	-0.1740	0.4272
Comorbidity	0.3690	0.0830
Treatment	0.1085	0.6221
Psychiatric admissions	0.4371	**0.0369**

Abbreviations: GAF = Global Assessment of Function. Bold values denote statistical significance, $p < 0.05$.

Table S20. Correlations between Levine´s clock "PhenoAge", sociodemographic and clinical characteristics.

Variable	R / rho	p value
Age	0.3508	0.1007
Schooling	0.1508	0.4920
GAF	-0.0541	0.8063
Comorbidity	0.2665	0.2188
Treatment	0.1011	0.6462
Psychiatric admissions	0.2073	0.3425

Abbreviations: GAF = Global Assessment of Function. Bold values denote statistical significance, $p < 0.05$.

Table S21. Correlations between Pediatric Buccal Epigenetic´s clock (PedBE), sociodemographic and clinical characteristics.

Variable	R / rho	p value
Age	0.4191	**0.0465**
Schooling	0.1375	0.5314
GAF	-0.5363	**0.0083**
Comorbidity	0.1662	0.4483
Treatment	0.2673	0.2175
Psychiatric admissions	0.6599	**0.0006**

Abbreviations: GAF = Global Assessment of Function. Bold values denote statistical significance, $p < 0.05$.

Table S22. Correlations between Wu´s clock, sociodemographic and clinical characteristics.

Variable	R / rho	p value
Age	0.3094	0.1508
Schooling	0.0900	0.6827
GAF	-0.4584	**0.0278**
Comorbidity	0.0835	0.7046
Treatment	0.3372	0.1156
Psychiatric admissions	0.6208	**0.0015**

Abbreviations: GAF = Global Assessment of Function. Bold values denote statistical significance, $p < 0.05$.

Table S23. Correlations between Zhang`s clock, sociodemographic and clinical characteristics.

Variable	R / rho	p value
Age	0.2479	0.2539
Schooling	0.0017	0.9937
GAF	-0.2150	0.3245
Comorbidity	0.2308	0.2893
Treatment	-0.0905	0.6812
Psychiatric admissions	0.4715	**0.0231**

Abbreviations: GAF = Global Assessment of Function. Bold values denote statistical significance, $p < 0.05$.
